# Sub-Luxation of Inferior Maxilla Backwards

**Published:** 1877-06

**Authors:** E. M. Moore


### ARTICLE V.

### *Sub-Luxation of the Inferior Maxilla Backwards.*

BY E. M. MOORE, JR., M. D.

Luxations of the jaw are described in the books by all the authors as occurring in two ways and in but one direction, viz: A complete luxation, where the condyle of the lower jaw has left the glenoid cavity and lies forward of the eminentia articularis and without the capsule. The other a sublaxation, which takes place in two ways, one where the capsular ligament, from some constitutional cause, has become elongated and will allow of a separation of the joint surface within the capsule, and the other from violence, where the end of the condyle, catching in front of the cartilage, crumpled it back, and is held forward of its normal position.

These are the conditions described in all the works on surgery. In vain have I searched the surgeries and journals to find anything different, and the accident, a report of which follows, is, as far as I can learn, unique.

Before reporting the case, I would like to give an outline of the anatomy of the joint, as it will assist me in the description of the accident, and the reasons for the diagnosis.

The glenoid fossa lies back of the middle root of the zygoma, which forms its anterior margin, and in front of the glasserian or glenoid fissure which limits its extent backwards. The cavity is oval in shape, the transverse being the long diameter. The anterior margin is rounded to allow of greater freedom of motion at the joint when the mouth is opened. The condyle of the lower jaw is also oval in shape, and the transverse diameter is also the longer.

It convex from side to side, and from before backwards, and in opening the mouth there is a peculiar condition present, that of convex surfaces opposing each other in the formation of a joint. In order to guard against the constant danger of dislocation of this joint, which would be present every time the mouth was widely opened, on account of this formation of the joint, there is interposed between the two joint surfaces an inter-articular-fibro cartilage, which conforms in general to the shape of the articulation, being convex posteriorly and slightly concave anteriorly and presenting a cup-shaped surface above and below which presents a concave surface to the convexity of the condyle and the anterior rounded margin of the glenoid ussa. The capsular ligament is deficient on the anterior and internal aspect of the joint to admit of the attachment of the external pterogoid muscle to the inter-articular-fibro cartilage. The joint is supplied with two synovial membranes.

Last August Mrs. H. came to Dr. Moore's office for relief, being unable to close her mouth or to open it but a little way without experiencing great pain at the left temporo maxillary articulation. The accident was caused by the patient biting off the superabundant stem of a rose which she held in her right hand. This accident had occurred several times before, as the following letter will show: Upon examination it was found that the lower jaw was separated from the upper one-quarter of an inch in front, and was drawn back three-eighths of an inch, measurement being made from the edge of the front teeth of the upper jaw to the anterior surface of the teeth of lower jaw when the mouth was closed as nearly as possible. The jaw was carried slightly to the left side. There was an appreciable difference felt in the relation of the condyle with the mastoid processes of each side. The saliva ran freely on the affected side.

The reduction was made by the operator standing behind the patient, the back of her head resting against his chest, and pressing up the chin with both his hands, a wedge of ivory having been previously placed between the teeth on the injured side, as far back as possible. The pressure was continued for two or three minutes, in order to stretch the capsul and smooth out the wrinkles. The wedge was then quickly removed, the pressure on the chin being continued. Upon releasing the patient it was found that the jaw had assumed its normal position, and the mouth could be closed and moved in any direction with but little pain. On measurement it was found that the lower jaw had come forward one-quarter of an inch.

The diagnosis made was sub-luxation backwards, on the following grounds: The position of the jaw, the relation of both jaws to each other, the restoration by the usual method of reduction and the cause of the accident. In biting off a thread or the stem of a rose, the motion of the jaw is peculiar. In performing this act the canine, or first bicuspid, the teeth are generally employed, and the motion of the jaw is from before backward, and generally from right to left, and at the same time the muscles which close the jaw are firmly contracted. In this case the condyle was carried just beyond the edge of the cartilage, and the firm contraction of the muscles crumpled the cartilage before it, and produced the sub-luxation.

Additional confirmation is desired, from the fact that in previous displacements reduction was accomplished by the extreme projection of the condyle forward, by the act of yawning.

---

ROCHESTER, March 1st, 1877.

DR. E. M. MOORE, DEAR SIR:—

The difficulty with my wife's mouth has been about as follows: Quite a number of years ago, when she was a young lady, she first noticed a tenderness where the jaws are united, with a difficulty in opening her mouth and in getting her front teeth together. This would happen occasionally, from what cause she does not now remember, and it always passed away in a day or two. Later she remembers frequently having caused the trouble by biting something hard with the side front teeth, and the difficulty would frequently pass off in a night, or she would get relief in *gaping*. She thinks she never was troubled more than two or three days at a time before you saw her. At that time she displaced her jaw by biting the stem of a rose which she had in her right hand, and it had been out of position for just a week. It appeared to be more completely out of joint than ever before, and she thinks would not have resumed its natural position without assistance.

Since then she had no serious trouble with it, though at times there has been felt a slight tenderness after biting too hard on something with the front teeth.

Mrs. H. thinks that nearly always the jaw was displaced in biting off her thread in sewing. Once a painful tap under the chin producing a similar effect.

Yours Respectfully, C. M. H.

—*Buffalo Med. and Surg. Journal.*

---

